# Supplementary material for: End Sequence Analysis Toolkit (ESAT) expands the extractable information from single-cell RNA-seq data
Source: Genome Res. 2016 Oct;26(10):1397–410. doi: 10.1101/gr.207902.116 (PMC5052061; doi:10.1101/gr.207902.116)
Supplement: Supplemental Material [file supp_gr.207902.116_Supp_Materials.tar.gz › ESAT-esat_v0.1/doc/html/classbroad_1_1core_1_1siphy_1_1tools_1_1conservation_1_1_estimate_omega_per_exon.html]

ESAT: broad.core.siphy.tools.conservation.EstimateOmegaPerExon Class Reference


ESAT


- Main Page
- Related Pages
- Packages
- Classes
- Files

- Class List
- Class Index
- Class Hierarchy
- Class Members

All Classes Namespaces Files Functions Variables Enumerator Pages

- broad
- core
- siphy
- tools
- conservation
- EstimateOmegaPerExon

Public Member Functions |
Static Public Member Functions |
List of all members

broad.core.siphy.tools.conservation.EstimateOmegaPerExon Class Reference

|  |  |
| --- | --- |
| Public Member Functions | |
|  | EstimateOmegaPerExon (File BEDFile, File modelFile, String alnDir, String alnFileFormat, String save) throws Exception |
|  | |
|  | EstimateOmegaPerExon (Collection< Gene > genes, File modelFile, String alnDir, String alnFileFormat, String save) throws Exception |
|  | |

|  |  |
| --- | --- |
| Static Public Member Functions | |
| static OmegaFit | fitOmega (Annotation align, File modelFile, String alnFile, String alnFileFormat) throws Exception |
|  | |
| static ArrayList< OmegaFit > | slideWindowComputeOmega (Annotation align, File modelFile, String alnFile, String alnFileFormat, int windowSize, int overlap) throws Exception |
|  | |
| static OmegaFit | fitOmega (Alignments align, TreeScaler scaler) throws Exception |
|  | |
| static ArrayList< OmegaFit > | slideWindowComputeOmega (Alignments align, TreeScaler scaler, int windowSize, int overlap) throws Exception |
|  | |
| static void | main (String[] args) throws Exception |
|  | |

## Constructor & Destructor Documentation

|  |  |  |  |
| --- | --- | --- | --- |
| broad.core.siphy.tools.conservation.EstimateOmegaPerExon.EstimateOmegaPerExon | ( | File | *BEDFile*, |
|  |  | File | *modelFile*, |
|  |  | String | *alnDir*, |
|  |  | String | *alnFileFormat*, |
|  |  | String | *save* |
|  | ) |  | throws Exception |

Here is the caller graph for this function:

|  |  |  |  |
| --- | --- | --- | --- |
| broad.core.siphy.tools.conservation.EstimateOmegaPerExon.EstimateOmegaPerExon | ( | Collection< Gene > | *genes*, |
|  |  | File | *modelFile*, |
|  |  | String | *alnDir*, |
|  |  | String | *alnFileFormat*, |
|  |  | String | *save* |
|  | ) |  | throws Exception |

## Member Function Documentation

|  |  |  |  |
| --- | --- | --- | --- |
| static OmegaFit broad.core.siphy.tools.conservation.EstimateOmegaPerExon.fitOmega | ( | Annotation | *align*, |
|  |  | File | *modelFile*, |
|  |  | String | *alnFile*, |
|  |  | String | *alnFileFormat* |
|  | ) |  | throws Exception |

static

|  |  |  |  |
| --- | --- | --- | --- |
| static OmegaFit broad.core.siphy.tools.conservation.EstimateOmegaPerExon.fitOmega | ( | Alignments | *align*, |
|  |  | TreeScaler | *scaler* |
|  | ) |  | throws Exception |

static

|  |  |  |  |  |  |
| --- | --- | --- | --- | --- | --- |
| static void broad.core.siphy.tools.conservation.EstimateOmegaPerExon.main | ( | String[] | *args* | ) | throws Exception |

static

Here is the call graph for this function:

|  |  |  |  |
| --- | --- | --- | --- |
| static ArrayList<OmegaFit> broad.core.siphy.tools.conservation.EstimateOmegaPerExon.slideWindowComputeOmega | ( | Annotation | *align*, |
|  |  | File | *modelFile*, |
|  |  | String | *alnFile*, |
|  |  | String | *alnFileFormat*, |
|  |  | int | *windowSize*, |
|  |  | int | *overlap* |
|  | ) |  | throws Exception |

static

|  |  |  |  |
| --- | --- | --- | --- |
| static ArrayList<OmegaFit> broad.core.siphy.tools.conservation.EstimateOmegaPerExon.slideWindowComputeOmega | ( | Alignments | *align*, |
|  |  | TreeScaler | *scaler*, |
|  |  | int | *windowSize*, |
|  |  | int | *overlap* |
|  | ) |  | throws Exception |

static

---

The documentation for this class was generated from the following file:

- java/broad/core/siphy/tools/conservation/EstimateOmegaPerExon.java


---

Generated on Fri Aug 15 2014 15:10:57 for ESAT by  

 1.8.7
